# ASO Author Reflections: Identifying and Understanding How Vulnerable Populations Are Affected by Delays in Pancreatic Cancer Care

**DOI:** 10.1245/s10434-024-15476-6

**Published:** 2024-06-07

**Authors:** John Fallon, Oliver Standring, Nandan Vithlani, Lyudmyla Demyan, Manav Shah, Emma Gazzara, Sarah Hartman, Shamsher Pasha, Daniel A. King, Joseph M. Herman, Matthew J. Weiss, Danielle DePeralta, Gary Deutsch

**Affiliations:** 1 Department of Surgical Oncology, Donald and Barbara Zucker School of Medicine at Hofstra/Northwell, Hempstead, NY USA; 2 Department of General Surgery, Northwell Health, New Hyde Park, NY USA; 3 Department of Surgical Oncology, Northwell Health Cancer Institute, Northwell Health, New Hyde Park, NY USA; 4 Division of Medical Oncology/Hematology, Northwell Health, New Hyde Park, NY USA; 5 Department of Radiation Oncology, Northwell Health Cancer Institute, Northwell Health, New Hyde Park, NY USA

## Past

As of 2022, the 5-year survival rate for pancreatic cancer in the United States remains dismal (5–15%), with a long-term survival rate of only 6%.^1^ Findings have shown that longer time to treatment of several cancers, including pancreas cancer, leads to an absolute greater risk of mortality ranging from 1.2 to 3.2% per week, highlighting the importance of effective, expeditious navigation of patients into the appropriate care tracts.^2^ The multiple steps required to diagnose and treat pancreatic ductal adenocarcinoma (PDAC) creates several opportunities for delay, which can highlight disparities in the care of certain populations. Due to the necessity for early detection, diagnosis, and initiation of treatment of PDAC to decrease mortality, it is important to understand the characteristics of patients presenting both in the outpatient setting and during emergent hospital admissions. This study aimed to both understand the patient characteristics and identify barriers to timely pancreatic cancer care in these populations.

## Present

This analysis initially aimed to uncover associations between the location of diagnosis in PDAC and patient characteristics and outcomes. The study found that underrepresented minorities (URMs) were 1.2 times more likely to have PDAC diagnosed during an emergency presentation (EP). Given recent studies showing that patients with cancer diagnosed in the emergency department have an increased mortality, the analysis evolved to highlight the patient characteristics of the EP population and its relationship to cancer treatment metrics.^3,4^ Subsequent analysis showed that regardless of diagnosis location, URMs had significantly longer times from presentation to biopsy and definitive treatment. Additionally, the study found that URMs waited an average of three times longer to receive a biopsy after initial presentation in the EP cohort and one week longer in the outpatient cohort than their non-URM counterparts. Overall, the findings showed that URMs are having significantly longer times to PDAC diagnosis and treatment, starting in both the inpatient and outpatient settings, than their non-URM counterparts. This delay can potentially be partially ameliorated by programs such as the authors’ pancreatic multidisciplinary clinic.^5^ These findings are important for improving PDAC care of URMs because it identifies when and where cancer care for these vulnerable populations is most likely being impeded.

## Future

Future efforts must highlight the need for health care systems to identify URM patients at increased risk for delayed times to diagnosis and treatment. One follow-up study to this analysis might aim to fully understand the denominator of new pancreas cancer diagnoses to elucidate the retention rate of URMs and identify areas in which to increase matriculation to definitive cancer care. Creating innovative solutions could include such policies as integrating pancreatic multidisciplinary programs into the inpatient hospital setting and emergency department to ensure that patients who present emergently and have positive imaging for PDAC are not discharged unless a biopsy has been performed. By streamlining care in the hospital setting, URMs who are more likely to present via this route could benefit greatly.
